# Bacteria-host relationship: ubiquitin ligases as weapons of invasion

**DOI:** 10.1038/cr.2016.30

**Published:** 2016-03-11

**Authors:** Timurs Maculins, Evgenij Fiskin, Sagar Bhogaraju, Ivan Dikic

**Affiliations:** 1 Institute of Biochemistry II, Goethe University Frankfurt, Theodor-Stern-Kai 7, 60590 Frankfurt am Main, Germany; 2 Fraunhofer Institute of Molecular Biology and Applied Ecology IME, Project Group Translational Medicine and Pharmacology TMP, Theodor-Stern Kai 7, 60596 Frankfurt am Main, Germany; 3 Buchmann Institute for Molecular Life Sciences, Max-von-Laue-Strasse 15, 60438 Frankfurt am Main, Germany

**Keywords:** bacterial effectors, ubiquitin ligases, bacteria, bacterial mimicry

## Abstract

Eukaryotic cells utilize the ubiquitin (Ub) system for maintaining a balanced functioning of cellular pathways. Although the Ub system is exclusive to eukaryotes, prokaryotic bacteria have developed an armory of Ub ligase enzymes that are capable of employing the Ub systems of various hosts, ranging from plant to animal cells. These enzymes have been acquired through the evolution and can be classified into three main classes, RING (really interesting new gene), HECT (homologous to the E6-AP carboxyl terminus) and NEL (novel E3 ligases). In this review we describe the roles played by different classes of bacterial Ub ligases in infection and pathogenicity. We also provide an overview of the different mechanisms by which bacteria mimic specific components of the host Ub system and outline the gaps in our current understanding of their functions. Additionally, we discuss approaches and experimental tools for validating this class of enzymes as potential novel antibacterial therapy targets.

## Introduction

Throughout evolution, prokaryotes have acquired an intricate array of molecular armory that facilitates the hijacking of host cells. In fact, a large portion of the bacterial genome is dedicated for mediating host infection — a central task in bacterial life cycle. The molecular details of this complicated process are now rapidly unfolding and clearly indicate the dependence of bacterial infection strategies on a number of 'toxins' or effectors that have various modes of action. Hijacking of the host cellular environment begins with a rapid rearrangement of actin cytoskeleton within the first 10-30 min of infection^1^. This abrupt change is brought about by bacterial effectors that activate small GTPases regulating the actin cytoskeleton, such as Rac1 and Cdc42^2,3^. As a result, membrane ruffling is enhanced thus mediating the increased internalization of bacteria via macropinocytosis. Invading bacteria take tight control of the activity of these cellular GTPases, as their prolonged activation would interfere with the bacterial life cycle by disturbing the cellular niche^4^. Once invasion has been successful, bacteria restore the cytoskeletal architecture by inactivating these cellular GTPases^5^.

In addition to its role in mediating bacterial internalization, macropinocytosis serves as a 'disguise' that helps bacteria to evade the host defence system. Through residing in modified phagosomal compartments, bacteria remain completely unnoticed by the host defence machinery. The phagosomal compartments have been especially well studied in the case of *Salmonella* infections, and have become known as *Salmonella*-containing vacuoles (SCVs). Takeover of cellular systems is facilitated by sophisticated mechanisms that coordinate the delivery of effectors into the host cytoplasm. Many Gram-negative plant and animal pathogens harbor secretion machineries, which are highly analogous to injection needles designed to penetrate membrane structures^6^. Although the impact of different secretion systems on bacterial virulence may vary, an essential role in pathogenicity is often attributed to the type III secretion system (T3SS)^7^. This complex injection needle consists of more than 40 proteins, many of which are conserved across plant and animal pathogens^8,9^. One of the best-described organisms in this regard is again *Salmonella*, which expresses two T3SSs encoded by *Salmonella* pathogenicity islands (SPI)-1 and -2. While T3SS-1 is required for active invasion, for example, to deliver effectors that mediate actin cytoskeleton rearrangements, expression of T3SS-2 promotes intracellular survival within SCVs. Following the early stages of infection, the T3SS-1 system is generally downregulated and the low pH environment and nutrient availability within SCVs triggers expression of T3SS-2^10^. In some cases, the exchange of the T3SS needles by *Salmonella* results in SCV membrane perforation and the release of a subpopulation of bacteria into the cytosol allowing their detection by the host defence system. In this case, the first line of host defence against infection is restriction of bacterial replication via a type of macroautophagy known as xenophagy^10^. Through this process intracellular bacteria that are not encapsulated in SCVs are sequestered in autophagosomes and their growth is further suppressed by degradation in the hydrolytic environment following lysosomal fusion. In the case of *Salmonella*, approximately one third of intracellular bacteria are targeted by autophagy one hour post infection^11^. *Salmonella*, however, does not need to be free in the cytosol for autophagic degradation to occur. In some instances, perforated vacuoles can also be targeted following binding to cellular galectins, which normally monitor endosomal and lysosomal integrity, resulting in recruitment of the autophagic machinery^12^. Interestingly, recent studies demonstrate that bacteria have evolved evasive techniques allowing them to escape autophagy and repair SCV membranes damaged by the T3SS-1 needle, therefore restoring the acidic environment within the vacuole and enabling expression of T3SS-2 and subsequent survival^13^.

Following T3SS-2 expression, bacterial cells are able to deliver the second wave of effectors that hijack signaling cascades of the host to promote invasion and dissemination of bacteria. Intriguingly, among the effectors that are injected by pathogens to the host cellular environment are proteins with ubiquitin (Ub) ligase activity^14^. Cellular ubiquitylation is proven to be one of the most elaborate post-translational regulation mechanisms developed by eukaryotic cells. It involves a coordinated action of at least three enzymes (E): Ub-activating E1, Ub-conjugating E2 and E3 Ub ligases^15^. There are two major types of E3 Ub ligases: (1) the RING type that function as scaffolds bringing E2s and the targeted substrates into proximity and (2) the HECT type that form a thioester intermediate with Ub before transferring it to the substrate^16^. This large class of enzymes plays a key role in ubiquitylation cascades by catalyzing the formation of iso-peptide bonds between the carboxyl terminus of Ub and target lysine (K) residues on the substrate protein^15^. Ub itself contains seven K residues, all of which could be used to form Ub chains *in vivo*^17^. Ub can also form linear chains, in which the carboxyl terminus of one Ub molecule forms a peptide bond with the amino terminus of another Ub^18^. In fact, distinct structural features of different poly-Ub chains define the recognition of ubiquitylated substrates by specific cellular receptors^19^. As a consequence, modification of cellular proteins by Ub signals is associated with varied physiological outcomes. For example, substrates modified by K48-linked chains are degraded by the proteasome, K63-linked chains, however, can play a signaling role in recruitment of DNA repair proteins, whereas linear Ub chains regulate the canonical nuclear factor (NF)-κB pathway that activates cytokine gene expression and the host inflammatory response^15,17,18,20^.

Prokaryotic bacteria do not possess genes encoding Ub. Nevertheless, some bacteria, such as *Mycobacterium tuberculosis*, harbor proteasomes and degrade their own protein substrates^21^. It is established that *Mycobacterium* possesses a functional analogue of the Ub system known as the prokaryotic Ub-like protein (Pup) system^22^. During pupylation, the Pup protein is covalently conjugated to K residues of protein substrates in a manner similar to Ub^23,24^. However, unlike the Ub system, which can mediate different physiological outcomes depending on the nature of ubiquitylation, the fate of pupylated substrates is restricted to proteasomal degradation. This is believed to be due to the highly unstructured nature of the Pup protein with a significant part of the protein lacking a stable secondary or tertiary structure^25,26,27^. Therefore, despite the overall functional similarity between the Pup and Ub systems, the intrinsically structurally disordered Pup may explain the inability of bacterial pathogens to utilize their Pup system as an effective modifier for manipulation of host signaling pathways. It is therefore likely that bacterial cells have evolved alternative routes, including the expression and release of Ub ligase-like effectors that are more compatible with the hosts' Ub machinery. As a consequence, bacteria are able to hijack the hosts' own Ub system, thereby manipulating a multitude of signaling cascades to promote bacterial survival.

In recent years it has become evident that bacterial Ub ligase-like effectors are central to the bacterial life cycle. Therefore, multiple studies have focused on identifying effector-specific host substrates in order to decipher key host signaling cascade modulations that are essential for bacterial survival and dissemination. To provide a better understanding of host-pathogen interactions, in this review we focus on known bacterial Ub ligases from different pathogens and describe their roles in modulating signaling cascades of the host. We also discuss the possibility of targeting these effectors for combating bacterial infections.

## Ub ligase mimicry in the 'arms race' between pathogen and host

*Pseudomonas syringae* is a pathogen of tomato and *Arabidopsis thaliana* cells and dedicates about 7% of its genome for producing effectors that dampen host innate immunity and promote disease in plants^28^. Plant cells, in turn, recognize the bacterial effectors released to the cellular environment through a process known as effector-triggered immunity (ETI)^29^. This type of response to infection leads to a localized immunity-associated programmed cell death (PCD), in which plants 'sacrifice' a limited portion of the leaf to protect the rest of the plant from a more severe systemic infection^30^. The interaction between *Pseudomonas* and its host involves a co-evolution of virulence effectors and the ETI pathway, which in many aspects could be depicted as a type of 'arms race'. Not surprisingly, bacterial Ub ligase-like effectors are at the heart of this battle. For example, the *Pseudomonas* AvrPtoB effector represents a mechanism by which bacteria suppress this conserved ETI pathway through inhibiting PCD activators^29^. The carboxy-terminal domain (CTD) of AvrPtoB is essential to avoid recognition by plant immunity, as deletion of this domain allows the amino-terminal region of AvrPtoB to be detected by certain tomato varieties leading to immunity-associated PCD^31^. The function and activity of the AvrPtoB CTD could not be predicted from initial examination of the primary amino acid sequence. It was only when Janjusevic and colleagues revealed the structure of the AvrPtoB CTD that it became apparent that it has a remarkable resemblance to the canonical eukaryote-specific RING domain present in Ub ligases^32^. This highly homologous domain serves as a binding site for E2 enzymes as mutations affecting this domain typically block E2 binding and impair Ub ligase activity^33^. Therefore, the presence of this conserved RING domain allows AvrPtoB to hijack the host Ub system for its own purposes, which is essential for bacterial virulence^31,32^.

Through recent molecular studies it has become apparent how host resistance components of the plant immune system enhance the capacity to recognize and respond to *Pseudomonas* infection. The Fen kinase directly interacts with the AvrPtoB amino terminus and is responsible for activating the plant ETI. Interestingly, the RING domain located in the CTD of AvrPtoB facilitates ubiquitylation and degradation of the Fen kinase, thereby counteracting ETI and increasing susceptibility of plants to disease^34,35^. Remarkably, the AvrPtoB-induced degradation of the Fen kinase is mediated through the host's own proteasomal system. Thus, in the case of the Fen kinase, by acquiring a Ub ligase activity, AvrPtoB has thwarted a highly conserved host resistance mechanism via 'turning' the host's proteasomal system against the host.

The 'arms race' between the host and the pathogen is further demonstrated by the interaction between AvrPtoB and another host target — the Pto kinase. Unlike the Fen kinase, this interaction favors the host defence system. By phosphorylating a regulatory threonine residue within the catalytic cleft of the RING domain of AvrPtoB, Pto diminishes the ligase activity of AvrPtoB and reduces ubiquitylation of its targets, including Fen — conferring Pto-mediated resistance against *Pseudomonas* infection^35^. Therefore, the outcome of *Pseudomonas* infection depends on a competition between the phosphorylation and ubiquitylation activities mediated by the host kinase and the invading effector ligase, respectively. Pto represents a host variant molecule with higher kinase activity and hence this defence countermeasure grants the host the ability to fight back against the pathogen.

## Ub ligase mimicry extends to mammalian pathogens

Plant pathogens are not unique in their ability to develop effectors that structurally or functionally mimic host Ub ligases to achieve their goals. Many human pathogens also utilize Ub ligase-like effectors that are secreted to the host cells (Table 1). One such pathogen, *Salmonella enterica*, can cause salmonellosis in humans, a disease commonly acquired by ingestion of contaminated food or water. Clinical syndromes of this infection include severe typhoid fever, caused by *Salmonella typhi* and *paratyphi* species, with fatality rate ranging between 10% and 20% in untreated cases and 1% following treatment with appropriate antibiotics. Additionally, a wide range of milder clinical syndromes have been described for non-typhoid *Salmonella*, which in healthy adults causes diarrhoeal disease with an untreated case fatality rate of 0.1%^36^.

*Salmonella* developed sophisticated mechanisms for effective invasion of macrophages and epithelial cells. Among the effectors that *Salmonella* delivers to the host are enzymes that possess Ub ligase activity and are implicated in suppressing the inflammatory host response^14^. One such effector is the SopA E3-like enzyme. Despite the lack of sequence similarity, the overall molecular structure of the SopA carboxyl terminus is highly reminiscent of the eukaryotic HECT domain with its characteristic architecture containing a two-lobe structure^37^. Much like host HECT Ub ligases, SopA utilizes a catalytic cysteine residue to form a Ub thioester intermediate, which is essential for its *in vitro* ubiquitylation activity^38,39^. Although there are no reported substrates to date, SopA is known to play a key role in the induction of enteritis. Intriguingly, both *sopA* insertion and deletion mutants demonstrate a 5-fold decrease in polymorphonuclear neutrophil transepithelial migration, a hallmark of *Salmonella* infection, highlighting the importance of SopA in mediating *Salmonella* pathogenicity^40^.

SopA shares 26% similarity with the NleL effector from enterohaemorrhagic and enteropathogenic *E*. *coli* strains (EHEC and EPEC, respectively)^38,41^. Similar to SopA, NleL is also delivered to the host's cytoplasm through the T3SS and has Ub ligase activity^38,42,43^. Importantly, the formation of attaching and effacing lesions, a hallmark of pathogenic *E*. *coli* infection following bacterial attachment to epithelial cells, is dependent on NleL^42^. Both SopA and NleL bacterial Ub ligases interact with host E2s in a manner similar to host E3s. By utilizing hydrophobic interactions between the conserved phenylalanine in the host UbcH7 E2 enzyme and the aromatic residues in SopA and NleL, these bacterial Ub ligase-like enzymes occupy the same region on the E2 as the host HECT E3s. This indicates that bacterial effectors may also interfere with cellular ubiquitylation by limiting the availability of cellular E2 enzymes.

**Figure 1:**
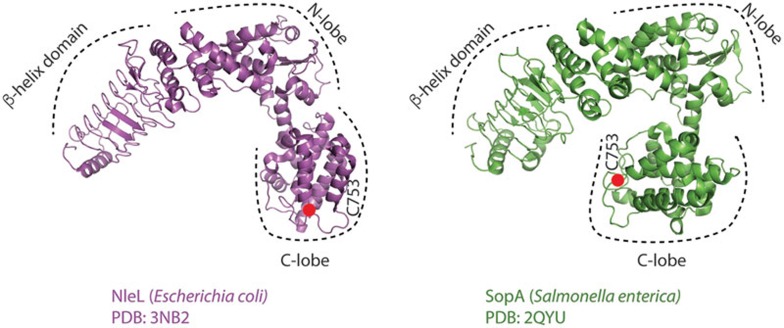
Comparison of the NleL and SopA crystal structures. Ribbon representations of the NleL (PDB code: 3NB2) and SopA (PDB code: 2QYU) HECT-like bacterial Ub ligases. Catalytic cysteine residues of NleL (C753) and SopA (C753) are represented as red spheres. The amino (N) and carboxyl (C) lobes of the HECT domain are indicated, as well as the amino-terminal substrate recognition β-helix domain.

Previous studies of human HECT ligases revealed that amino-terminal extensions upstream of the HECT domain determine substrate recognition. For this purpose, human HECT Ub ligases contain a variety of domains that facilitate substrate recognition and protein-protein interactions^44^. The domain architecture of bacterial SopA and NleL enzymes is also organized in a similar fashion. The HECT-like domain is located at the carboxyl terminus with the amino-terminal part of SopA and NleL containing a parallel β-helix domain that is hypothesized to serve as a substrate-binding region^38,39^ (Figure 1). Interestingly, the C-lobes of both SopA and NleL HECT-like domains possess a high degree of conformational flexibility, indicating that bacterial Ub ligases harbor the necessary structural organization that allows them to facilitate Ub transfer from E2s onto the targeted substrates, similarly to eukaryotic HECT E3s^39^.

As indicated, SopA and NleL are crucial for mediating *Salmonella*-induced enteritis and *E*. *coli* lesions, respectively. This calls for the identification of their specific host targets, which is promising to unveil key host pathways modulated by these Ub ligases involved in enhancing bacterial pathogenicity. This information, in addition to increasing our understanding of host-pathogen interactions, can also provide insight into novel therapeutic approaches, as discussed later, to combat bacterial infection.

**Figure 2:**
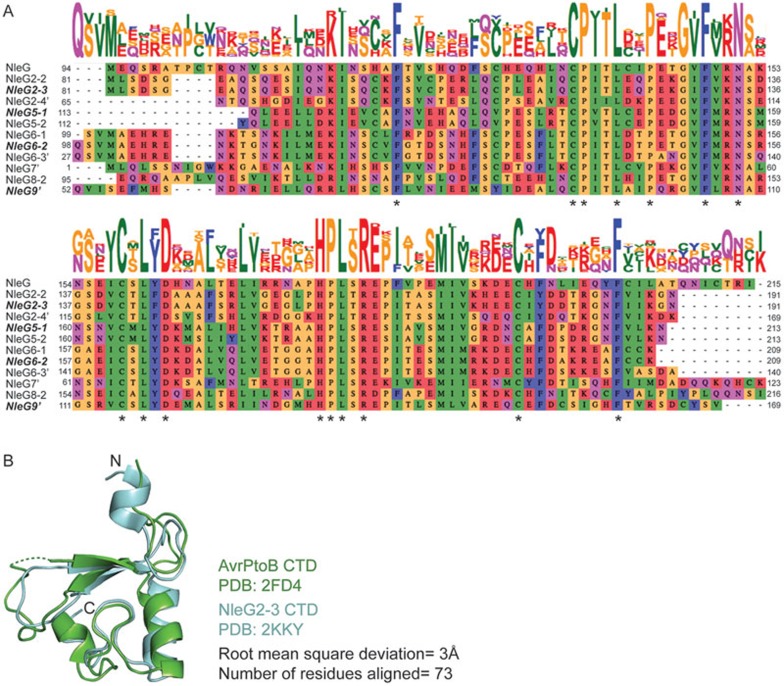
The carboxyl terminus of NleG effectors harbors RING/U-box domain. **(A)** Amino acid sequence alignment of NleG family member carboxy-terminal domains as analyzed by Clustal Omega^49,50^. Conserved amino acids corresponding to the RING/U-box motif are denoted with asterisks. NleG family members with demonstrated Ub ligase activity *in vitro* are highlighted in bold italic^48^. **(B)** Comparison of RING/U-box-like domains of AvrPtoB and NleG2-3. CTD structures of AvrPtoB (PDB code: 2FD4) and NleG2-3 (PDB code: 2KKY) were superimposed using Dalilite server^51^. For clarity in comparison, peripheral elements of the CTDs are removed from both structures, showing only the core of the RING/U-box domains.

Among the largest groups of bacterial effectors is the family of NleG proteins that are present in a variety of pathogenic EHEC and EPEC strains. NleG effectors are secreted via the T3SS system and have been linked to lethal *E. coli* infections^43,45^. Comparative sequence analysis of NleG family members revealed a highly conserved region of ∼100 amino acids in the CTD, which is annotated as the domain of unknown function (DUF) 1076. Interestingly, the NMR structure of NleG2-3 CTD, an NleG family member, uncovered the presence of a motif similar to the eukaryotic RING/U-box domain, which is a modified version of the RING domain that confers Ub ligase activity^46,47,48^. Almost all NleG effectors contain this conserved motif embedded in the sequence of their CTDs, suggesting that they function through host Ub ligase mimicry to enhance pathogenicity (Figure 2A)^49,50^. In fact, *in vitro* Ub ligase activity has been reported for some family members, including NleG2-3, NleG5-1, NleG6-2 and NleG9^48^. Remarkably, the tertiary structure of the NleG2-3 CTD is strikingly similar to that of the *P. syringae* AvrPtoB CTD, despite low amino acid sequence identity^51^ (Figure 2B). This provides additional evidence supporting a potential role of NleGs as effectors with Ub ligase activity inside the host cellular environment.

In contrast to the highly conserved CTDs, the amino termini of NleGs have low sequence identity and do not harbor any functional domains that can hint on how the cellular targets are recognized. Attempts to identify protein-protein interactions of some NleG effectors using the yeast two-hybrid (Y2H) technology were unsuccessful, despite finding novel high-confidence interacting partners of other effectors of pathogenic *E. coli* in the same screen^52^. Therefore, at present, mechanisms by which NleGs modulate host signaling are not well understood.

*Legionella* *pneumophila* utilizes yet another strategy that is based on mimicking functional domains involved in host Ub signaling. *Legionella pneumophila* invades and establishes a replicative niche in human macrophages by residing in *Legionella*-containing vacuoles (LCVs)^53^. In addition to delivering effectors containing U-box motifs that hijack host E2s to mediate target ubiquitylation^54,55^, *Legionella* also injects a family of F-box-containing proteins^56^. By utilizing the conserved F-box motif, these effectors interact with the Skp1 subunit of host multimeric Ub ligase complexes known as SCF (Skp1-Cullin-F-box) and act as adaptors that can recruit substrates for ubiquitylation^56^. For example, the AnkB effector of *Legionella* is anchored to the cytosolic face of the LCVs, where this effector mediates K48-specific ubiquitylation of proteins through the host's SCF complex^56,57,58^. By inducing degradation of proteins *en mass* AnkB satisfies the high demands of *Legionella* for amino acids and thus acts as a crucial weapon for promoting intracellular growth of this pathogen^57^.

## Bacteria further diversify the armory of Ub ligases

**Figure 3:**
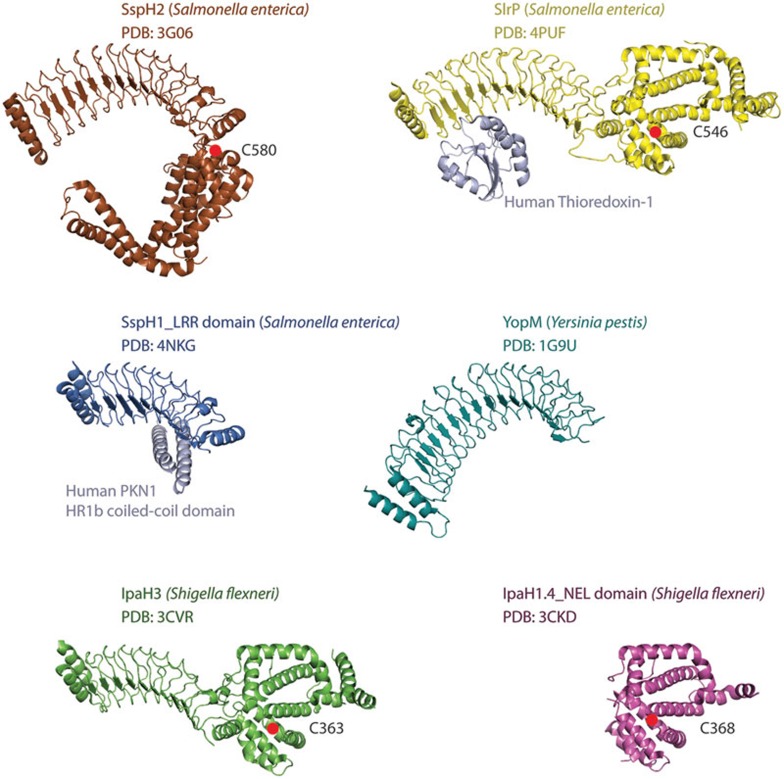
Structures of NELs from various pathogens. Ribbon representations of full-length NEL proteins (SspH2, SlrP, YopM, IpaH3), LRR (SspH1) and NEL (IpaH1.4) domains from various pathogens. *Salmonella enteric* SlrP and SspH1 structures are depicted in substrate-bound states. Catalytic cysteine residues of SspH1 (C580), SlrP (C546), IpaH3 (C363) and IpaH1.4 (C368) are represented as red spheres.

Structural or functional mimicry of the host Ub system has apparently proven to be an effective strategy for manipulating host signaling. In addition to acquiring effectors with RING and HECT-like domains, bacterial pathogens further diversified the armory of Ub ligases by evolving enzymes that are structurally distinct from their mammalian counterparts, termed NELs (novel E3 ligases). NELs constitute a family of at least 15 enzymes that spans across 6 pathogenic bacterial genera, including highly pathogenic *Salmonella* and *Shigella*^59^. NELs harbor three essential E3 ligase elements. First, the E2-interacting surface residues are involved in hijacking host E2s charged with Ub. Second, although NELs lack the *bona fide* bi-lobal HECT domain, these bacterial ligases have a catalytic cysteine residue utilized for Ub transfer. Last, the presence of leucine-rich repeats (LRRs) of variable length ensures the recognition of a wide array of targets (Figure 3)^60,61,62,63,64^.

*Shigella flexneri* IpaH effectors were the first described NELs. *Shigella* encodes at least three IpaH ligase effectors on its virulence plasmids and seven additional effectors are encoded by its genomic DNA^65,66,67,68^. All of IpaH effector proteins are secreted via the T3SS and thus potentially play a role in pathogenicity^69^. The IpaH9.8 ligase (encoded by a 9.8 kb DNA fragment on the virulence plasmid) was found to disrupt the pheromone response through supressing the MAPK pathway by promoting proteasome-dependent degradation of the MAPK kinase Ste7 when overexpressed in budding yeast^59^. Although IpaH9.8 E3 Ub ligase activity has been reported *in vitro*, it is still not clear whether IpaH9.8 affects the MAPK pathway in mammalian cells in a similar manner. IpaH9.8 also attenuates NF-κB signaling by degrading NEMO (NF-κB essential modulator), an essential component of this signaling cascade^70^. Mice infected with *Shigella* expressing a catalytically inactive IpaH9.8 C337A mutant exhibited reduced bacterial multiplication with an augmented inflammatory response as compared with mice infected with the wild-type strain^70^. Another member of the IpaH ligase family, IpaH7.8, was also reported to have Ub ligase activity *in vitro* and has been shown to facilitate the escape of virulent bacteria from endocytic vacuoles in mouse and human macrophages^59,71^. Control mice infected with wild-type *Shigella* exhibited enhanced bacterial multiplication as compared to mice infected with mutant bacteria lacking IpaH7.8. IpaH7.8 was also suggested to play a central role in promoting macrophage cell death through the activation of inflammasomes. Glomulin, a natural inflammasome inhibitor, is targeted by IpaH7.8 for degradation, which in turn stimulates inflammasome activation, macrophage cell death and dissemination of bacteria^72^. Another example of the role of the IpaH family in modulating the host's inflammatory pathways is IpaH0722 that was demonstrated to ubiquitylate and mediate the proteasomal degradation of TRAF2, thereby inhibiting protein kinase C-mediated NF-κB pathway activation upon *Shigella* infection^73^. Additionally, in a Y2H screen the NEL IpaH4.5 was identified to interact with the NF-κB transcription factor subunit p65 and was shown to drive p65 ubiquitylation and to inhibit TNFα-mediated NF-κB activation *in vitro*^74^.

In addition to the NELs described above, the role of NELs in promoting bacterial pathogenicity is further demonstrated in *Salmonella* infection. *Salmonella* encodes three members of the NEL family: SspH1, SspH2 and SlrP. All three members were shown to contribute to virulence in animal models of infection by mechanisms that are currently not well understood. SlrP mutant bacteria show colonization defects upon infection of mice, whereas both SspH1 and SspH2 are required for the induction of lethal infections in calves^75,76^. Using a Y2H screening approach, both Thioredoxin-1 and the host chaperone ERdJ3 were identified as interacting proteins of SlrP^77,78^. These data implicate SlrP in the inhibition of the overall stress response upon bacterial infection, yet more studies are required to dissect the exact mode of action of SlrP.

Once inside the host cell, SspH1 localizes to the nucleus, inhibits NF-κB-dependent gene expression and contributes to regulation of pro-inflammatory cytokine production. The SspH1 LRR domain was shown to bind protein kinase PKN1 in Y2H experiments^79^ and ubiquitylate PKN1 *in vitro*^59^. Initial affinity purification mass spectrometry (AP-MS) efforts identified the NOD receptor co-chaperone Sgt1 as an SspH2-binding partner. Subsequently, SspH2 was also demonstrated to enhance NOD1-mediated responses in cells. Mechanistic studies show that SspH2 stimulates the non-proteolytic ubiquitylation of NOD1 in heterologous expression experiments leading to agonist-independent activation of signaling^80,81^.

Structural studies provide interesting insight into the regulation of NEL ligase activity^64^. It appears that in the absence of physiological substrates, the catalytic cysteine residue is structurally occluded by carboxy-terminal LRRs, resulting in autoinhibition of NELs. This autoinhibitory mechanism is reminiscent of mammalian HECT Ub ligases. For example, autoinhibition of Smurf2 catalytic activity by its C2 domain is suggested to reduce spurious ligase activity^82^. Although the purpose of NEL autoinhibition is still unclear, it is possible that this regulation is important for restricting their activity to avoid autoubiquitylation and subsequent degradation or simply to minimize detection by the host. NEL autoinhibition might also function as an additional regulatory step to prevent competition with other bacterial ligases for host E2s in situations where NEL host substrates are absent. As with other bacterial Ub ligase-like effectors, the full spectrum of ubiquitylated substrates by NELs is currently unknown. Moreover, a global overview of the ubiquitylated host proteome by these effectors and knowledge on the dynamics of substrate ubiquitylation during infection are currently lacking.

In addition to *Shigella* and *Salmonella* species, pathogenic *Yersinia* species also encode the YopM effector that is required for full virulence in mouse infection models^83^. Although YopM contains a number of LRRs in its carboxyl terminus and its amino terminus was suggested to harbor similarity to the NEL domain of *Salmonella* and *Shigella* effectors, a Ub ligase activity for this effector has not been reported^84,85^. Instead, YopM serves as a scaffolding protein that facilitates the formation of a novel complex between RSK and PRK host kinases that normally do not interact^86^. Although this results in stimulation of kinase activity, at present the molecular role of the YopM/RSK/PRK complex remains unknown^87^. YopM also directly inhibits caspase-1 function in infected macrophages, which is essential for *Yersinia pseudotuberculosis* virulence *in vivo*^88^.

## Bacterial Ub ligase-like effectors as potential drug targets

The examples of bacterial Ub ligase-like effectors described above illustrate an important role of these enzymes in modulating host cellular signaling, thus facilitating infection and dissemination of pathogens. Although much remains to be learnt about different classes of bacterial Ub ligase-like enzymes and their mechanisms of action, it is important to consider the pharmacological inhibition of their activity as a novel strategy for treating severe pathogenic bacterial infections. Ub ligase drug discovery is minimal compared to other classes of signaling molecules, such as protein kinases or G protein-coupled receptors^89^. This is mainly due to limited tools currently available for developing robust high-throughput screening assays against Ub ligases of interest. The absence of ATP-binding pockets within the RING and HECT catalytic domains presents further challenges associated with targeting protein-protein interactions between Ub ligases and their cognate substrates or upstream E2-conjugating enzymes. Despite these limitations, a number of approaches, as described below, could potentially be utilized for targeting bacterial Ub ligase-like enzymes.

During the ubiquitylation reaction Ub ligases are positioned in close proximity to substrate proteins, which enables the application of the fluorescence resonance energy transfer (FRET) technology^90,91^. Provided that efforts in identifying high-confidence substrates of bacterial Ub ligase-like effectors will be intensified in the future, one could envision developing robust assays for targeting the interaction between Ub ligases and their cognate substrates utilizing FRET or similar *in vitro* reconstitution assays^92^. Structure-driven approaches also offer powerful tools in identifying potent inhibitors of Ub ligases^93,94^. Therefore, it is important to continue obtaining high-resolution structures of protein-protein interactions between bacterial Ub ligase-like enzymes and their substrates or partner E2s. Substrate-independent *in vitro* assays have also been designed for developing inhibitors of autocatalytic activity of physiologically relevant Ub ligases^95^. Since the catalytic activity of NELs plays an important role in the life cycle of a number of human pathogens and the conserved bacterial NEL domain appears distinct from known mammalian Ub ligases, the development of a broad-spectrum 'pan-NEL' inhibitor could potentially offer unmatched therapeutic benefits in multiple disease areas. More recently, pharmacological targeting of Ub ligase activity has been advanced to cellular assays^96^. Adopting a similar approach might therefore enable the generation of small-molecule inhibitors against the NEL active site within the host cellular environment. Since NELs are unique to bacteria, one could anticipate a very low cross-reactivity of such inhibitors against the host Ub ligases thereby potentially reducing non-specific side effects.

## Concluding remarks

The power of Y2H technology in allowing the fast interrogation of binary interactions between bacterial Ub ligase-like effectors and host substrates on a proteome-wide scale was instrumental for the identification of numerous host-pathogen interactions^97,98^. However, this technology also harbors several limitations, such as the use of yeast as a surrogate organism for mimicking the environment of the mammalian cell and the difficulty in detecting interactions involving multiple binding partners. In this context, AP-MS approaches provide a valuable complementary technology for the analysis of protein interaction networks and may also prove to be essential for the identification of physiological targets of bacterial Ub ligase-like enzymes. Of utmost importance is the development of methodology enabling the identification of proteins that are specifically ubiquitylated upon infection of host cells by various pathogens, which should significantly improve the identification of high-confidence targets that are relevant for bacterial infection. In addition, given that target phosphorylation plays an important role in substrate recognition by Ub ligases^99^, it is also important to identify host cellular kinases that may participate in target recognition by bacterial E3s. Furthermore, the continuous advancements in our understanding of target selectivity and activity of bacterial Ub ligases-like effectors necessitate the development of novel research tools. Activity-based probes (ABPs) harboring chemical warheads that covalently bind to the active site of enzymes have been previously developed for monitoring the activity of the Ub system^100,101^. Recent improvements of ABPs made it possible to detect the activity of host Ub ligases^102^. Therefore, ABPs could potentially represent useful tools for monitoring the activity of bacterial Ub ligase-like enzymes and could also be utilized in pharmacological screening to search for a more effective antibacterial therapy.

In conclusion, given the complexity of the host-pathogen 'arms race', it is instrumental that we combine the power of multiple techniques to tackle the different aspects of host-pathogen interactions. This promises to vastly expand our current knowledge and provide a robust foundation for identifying new tools to combat bacterial infection.

## Figures and Tables

**Table 1:** Known Ub ligase-like effectors of human bacterial pathogens

Effector	Pathogenic bacterial species	Target/Substrate	Function
**HECT-like**			
SopA	*Salmonella*	Unknown	Regulation of host inflammation
NleL	*EHEC*	Unknown	Regulation of actin pedestal formation
**RING/U-box-like**			
NleG family members	*EHEC/EPEC*	Unknown	Unknown
LubX	*Legionella*	CLK1, SidH	Promotion of growth in macrophages
**F-box**			
AnkB	*Legionella*	K48 poly-ubiquitylated proteins	Acquisition of nutrients
LegU1	*Legionella*	BAT3	Unknown
**Novel E3 ligases**			
SlrP	*Salmonella*	TXN, ERdj3	Regulation of cell death
SspH1	*Salmonella*	PKN1	Inhibition of androgen receptor and NF-κB signaling
SspH2	*Salmonella*	SGT1, NOD1	Activation of NOD-mediated NF-κB signaling
IpaH9.8	*Shigella*	NEMO, U2AF53	Inhibition of NF-κB signaling and inhibition of splicing
IpaH7.8	*Shigella*	Glomulin	Promotes macrophage cell death via inflammasome activation
IpaH4.5	*Shigella*	p65	Inihbition of TNFα-mediated NF-κB signaling
IpaH0722	*Shigella*	TRAF2	Inihibition of PKC-mediated NF-κB signaling
IpaH family members	*Shigella*	Unknown	Unknown
SidC/SdcA	*Legionella*	Unknown	Recruiting ER vesicles to LCV
